# Evaluation of the efficacy and safety of olanzapine as an adjunctive treatment for anorexia nervosa in adolescent females: a randomized, double-blind, placebo-controlled trial

**DOI:** 10.1186/1471-2431-8-4

**Published:** 2008-01-31

**Authors:** Wendy Spettigue, Annick Buchholz, Katherine Henderson, Stephen Feder, David Moher, Kader Kourad, Isabelle Gaboury, Mark Norris, Sheila Ledoux

**Affiliations:** 1 Mental Health Department, Children's Hospital of Eastern Ontario Research Institute, 401 Smyth Rd, Ottawa, ON, K1H 8L1, Canada; 2 Department of Psychiatry, University of Ottawa, 550 Cumberland, Ottawa, ON, K1N 6N5, Canada; 3 Department of Psychology, Carleton University, 1125 Colonel By Drive, Ottawa, ON, K1S 5B6, Canada; 4 Department of Pediatrics, Children's Hospital of Eastern Ontario, 401 Smyth Rd, Ottawa, ON, K1H 8L1, Canada; 5 Chalmers Research Group, 401 Smyth Rd, Ottawa, ON, K1H 8L1, Canada; 6 Department of Epidemiology and Community Medicine, Faculty of Medicine, University of Ottawa, 550 Cumberland, Ottawa, ON, K1N 6N5, Canada; 7 Clinical Research Unit, Children's Hospital of Eastern Ontario Research Institute, 401 Smyth Rd, Ottawa, ON, K1H 8L1, Canada

## Abstract

**Background:**

Anorexia Nervosa (AN) is a serious, debilitating condition that causes significant physical, emotional, and functional impairment. The condition is characterized by destructive weight loss behaviours and a refusal to maintain body weight at or above a minimally normal weight for age and height. AN often develops in adolescence and is a predominantly female disorder. Treatment for AN typically involves medical, nutritional and psychological interventions. Pharmacotherapy is also often used; however, the literature on the effectiveness of these drugs in a pediatric population is very limited. Olanzapine, which is an 'atypical' antipsychotic, is becoming more widespread in the treatment of AN. Olanzapine is hypothesized to facilitate weight gain, while decreasing levels of agitation and decreasing resistance to treatment in young women with AN. This randomized, double-blind placebo-controlled trial seeks to examine the effectiveness and safety of olanzapine in female youth with AN.

**Methods/Design:**

Adolescent females between the ages of 12 and 17 diagnosed with AN (either restricting or binge/purge type) or Eating Disorder Not Otherwise Specified with a Body Mass Index of less than or equal to 17.5, will be offered inclusion in the study. Patients will be randomly assigned to receive either olanzapine or placebo. Patients assigned to receive olanzapine will start at a low dose of 1.25 mg/day for three days, followed by 2.5 mg/day for four days, 5 mg/day for one week, then 7.5 mg/day (the target dose chosen) for 10 weeks. After 10 weeks at 7.5 mg the medication will be tapered and discontinued over a period of two weeks. The effectiveness of olanzapine versus placebo will be determined by investigating the change from baseline on measures of eating attitudes and behaviors, depression and anxiety, and change in Body Mass Index at week 12, and after a follow-up period at week 40. It is anticipated that 67 participants will be recruited over two years to complete enrollment.

**Discussion:**

Randomized controlled trials designed to measure the safety and effectiveness of olanzapine in comparison to placebo are desperately needed, particularly in the adolescent population.

**Trial registration:**

Current Controlled Trials ISRCTN23032339

## Background

The current knowledge of Anorexia Nervosa (AN) indicates that it is a complex, serious, and often-chronic condition characterized by an interplay of debilitating cognitive, emotional and physical processes, and destructive weight loss behaviors. The diagnostic criteria for AN consists of a refusal to maintain body weight at or above a minimally normal weight for age and height; an intense fear of gaining weight or becoming fat; a disturbance in the way in which one's body weight or shape is experienced, and in post-menarchal females, the absence of at least three consecutive menstrual cycles [1]. AN often develops in adolescence and is found to be most prevalent in youth, with the age of onset typically peaking between 15 and 19 years [2]. Lucas and colleagues [2] reported incidence rates of AN in the northern United States to be 25.7 females and 3.7 males per 100,000 of the population per year in the 10 to 14 year old age group, and 69.4 females and 7.3 males with AN per 100,000 of the population per year in the 15 to 19 year old age group. Currently, at the Children's Hospital of Eastern Ontario (CHEO), we treat on average 60 new eating disorder patients per year, 20 of whom would require hospitalization due to medical instability from AN.

Development of AN predisposes children and adolescents to a number of potentially harmful physical and emotional sequelae. Adverse physical effects related to profound weight loss and starvation include bradycardia, hypotension, hypothermia, dehydration, cardiac arrhythmias, heart failure, kidney failure, pancreatitis, amenorrhea, osteopenia, neurological impairments, and hormonal imbalances [3]. Prolonged illness can also result in impairments in long-term growth, bone density and fertility [4]. Psychological sequelae of AN include depression, suicidality, mood lability, social withdrawal, cognitive impairment, insomnia, irritability, agitation, and marked deterioration in family relationships [5]. Sullivan [6] reported mortality rates in individuals with eating disorders that exceeded the expected incidence of death from all causes in women 15 to 24 years of age by 12-fold. Many adolescents diagnosed with AN have difficulties functioning at school or with their peers resulting in increased isolation and social withdrawal.

Current treatment of AN involves a multidisciplinary approach. This includes medical and nutritional rehabilitation as well as psychological interventions [7]. There have not been any large randomized trials showing efficacy of any one pharmacological agent for the treatment of AN to date. A recent prospective study suggested that eating disorders are very difficult to treat with only 30% to 50% of patients fully recovering from the illness after six years [8]. There is evidence to suggest that patients that present with an extended preceding history of anorexic behaviour are more difficult to treat and cure and that early interventions improve prognosis [5]. Psychotropic medications such as antidepressants and antipsychotics have been investigated for the treatment of eating disorder patients either in addition to or instead of psychologically based treatment [9]. A recent review on the use of pharmacotherapy in the treatment of AN indicated that antidepressant use showed no benefit when compared to standard care [10]. Hence, antidepressant medications are not recommended as a first line treatment for AN, although there is some limited evidence suggesting a potential role for antidepressants in maintenance treatment of weight-recovered patients [11]. Antipsychotic medications such as chlorpromazine and pimozide have also been investigated as adjunctive treatment options for AN [10]. These medications have been associated with significant short-term and long-term side effects, and have not been found to be helpful in changing attitudes towards eating behaviour or body image. Long-term improvements in outcome in those treated with these medications are also lacking. For these reasons, the risks of using these particular medications are felt to outweigh any potential benefit. [10,12]

More recently, newer "atypical" antipsychotic medications such as olanzapine have been investigated as a possible treatment option for patients with AN. Olanzapine is a thienobenzodiazepine which has been studied extensively for the treatment of schizophrenia, and which more recently has been indicated for the treatment of bipolar disorder. Its mechanism of action is assumed to lie in its ability to block dopaminergic and serotonergic receptors (D1-4 antagonism and 5 HT 2A/2C antagonism). Small case reports and case series have shown some evidence of success using this medication for the treatment of AN; success in the short term being indicated bypatients increased BMIs, decreased levels of anxiety regarding food, and a decrease in ruminating thoughts surrounding food and body concerns[13,14]. Additionally, the side effect profile of atypical antipsychotics such as olanzapine may be more favorable than older antipsychotics [12]. Antipsychotic medications are hypothesized to reduce anxiety and agitation, as well as mitigating obsessions or delusions regarding weight and body shape [15]. Moreover, these types of medications have been linked to increased weight gain in other psychiatric populations, although the exact mechanism for this remains unclear [16,17].

In recent reviews of pharmacology and eating disorders, antipsychotic medications have demonstrated promise in the treatment of eating disorders. In spite of reported clinical success [14,18-20], and open trials: [21,22]), we are only aware of two published randomized controlled trials (RCT) which have evaluated the effectiveness of olanzapine [23,24]. The first of RCT contained a very small sample of adult patients with AN and compared the effects of olanzapine against chlorpromazine in reducing anorexic patients ruminating thoughts. Olanzapine was shown to be more effective than chlorpromazine in decreasing ruminative anorectic cognitions [23]. The second RCT investigated the effects of adding olanzapine treatment (randomized double blinded design) to patients participating in cognitive behavioral therapy. The number of patients in this study was also low, but psychological improvements were significant in the olanzapine group compared to the control group in specific cognitive domains. BMI's also significantly increased in the anorexic population with binge/purge subtype compared to the control group [24]. In recent case reports, olanzapine has been associated with considerable weight gain and maintenance, clinically notable decreases in levels of agitation, and improvements in sleep [14,18], general functioning, and overall compliance with treatment in children and adolescents diagnosed with AN [18,25,26].

Despite the lack of much empirical evidence, antipsychotic medications such as olanzapine are now widely used in the treatment of severe eating disorders. Recent case reports have demonstrated that olanzapine may be associated with marked improvements in weight gain, along with decreased anxiety and agitation, decreased obsessionality, and decreased resistance to treatment [14,18-22,25,26]. However, olanzapine will remain classified as an 'experimental' drug rather than a recommended and accepted treatment for AN, until safety and efficacy can be established in this population using a high quality randomized trial.

It is hypothesized that adolescents who are diagnosed with a severe eating disorder and are treated with olanzapine will demonstrate reduced disordered eating attitudes and behaviours as well as an increased rate of weight gain, as compared to a control group treated with placebo. It is also hypothesized that patients treated with olanzapine will demonstrate better long-term clinical outcomes in comparison to patients treated with placebo. It is also predicted that the adverse effects of olanzapine will be minor given the relatively low dose (as compared to treatment for patients with schizophrenia), slow titration, and short-term use of olanzapine.

## Methods/Design

This study is a randomized, double-blind, placebo-controlled trial. The ethics committee at the Children's Hospital of Eastern Ontario has approved the study protocol.

### Study Population

Adolescent females living within Eastern Ontario, Canada, who are between the ages of 12 and 17 and have been diagnosed with AN (either restricting or binge/purge type) or Eating Disorder Not Otherwise Specified (EDNOS) with a BMI of less than or equal to 17.5, will be offered participation in the study. Adolescents with a diagnosis of EDNOS and a low BMI are included as it is recognized that though not necessarily meeting all of the criteria for Anorexia Nervosa (see inclusion criteria), these two diagnoses are conceptualized similarly in terms of development and treatment.

### Trial Design

A randomized, double-blind, placebo-controlled design will be used to determine the efficacy and safety of olanzapine in treating AN. Consecutive female patients diagnosed with a severe eating disorder, who meet eligibility criteria, and who consent to participate in the study, will be randomly assigned to one of two groups: olanzapine (OLA) or placebo (PLA). A medication schedule will be in place for a total of 14 weeks (see Table 1 for Schedule of Events). Medication will be started at a low dose and increased up to a predetermined maximum dose, or until an intolerable side effect occurs. Both groups will receive either olanzapine or placebo for a total of 14 weeks. Both groups will continue to receive standard care that includes psychological, nutritional, and medical treatment.

**Table 1:** Schedule of Events

Timelines + 3 days	Day -7	Day 0	Day 7	Day 14	Day 21	Day 28	Day 35	Day 42	Day 49	Day 56	Day 63	Day 70	Day 77	Day 84	Day 91	Day 98	Day 105	Day 280+ 7
VISIT	Screen	Week 0 Rand	Week 1	Week 2	Week 3	Week 4	Week 5	Week 6	Week 7	Week 8	Week 9	Week 10	Week 11	Week 12	Week 13	Week 14	Week 15 or Term	Week 40 F/UP

Informed Consent	x																	
Medical History	x																	
Demographics	x																	
Inclusion/Exclusion	x	x																
Height	x													x				x
Weight & BMI score	x	x	x	x	x	x	x	x	x	x	x	x	x	x	x	x	x	x
Physical Exam	x																x	
Randomization		x																
Vital Signs	x	x	x	x	x	x	x	x	x	x	x	x	x	x	x	x	x	x
Hematology & Chemistry*	x			x		x				x				x			x	
HBA1C				x													x	
Multistick Urinalysis	x	x	x	x	x	x	x	x	x	x	x	x	x	x	x	x	x	
Urine for Pregnancy	x	x	x	x	x	x	x	x	x	x	x	x	x	x	x	x	x	
Electrocardiogram (EKG)	x			x		x											x	
Adverse Events			x	x	x	x	x	x	x	x	x	x	x	x	x	x	x	x
Concomittant Meds (mg)	x	1.25 → 2.5	5	7.5	7.5	7.5	7.5	7.5	7.5	7.5	7.5	7.5	7.5	5.0	2.5	0	0	0
Dispense Study Med		x	x	x	x	x	x	x	x	x	x	x	x	x	x			
AIMS			x	x	x	x	x	x	x	x	x	x	x	x	x	x	x	
Psychological Measures																		
EAT-26		x						x						x			x	x
CDI **		x						x						x			x	x
MASC **		x						x						x			x	x
CAPI		x						x						x			x	x
CBCL**		x						x						x			x	x
EDS3		x						x						x			x	x

*namely CBC and diff, electrolytes, Mg, Ca, P04, BUN, Cr, fasting glucose, Albumin, Ferritin, vit.B12, RBC folate, FSH, LH, estradiol, prolactin, βHCG, TSH, INR, cholesterol and triglycerides, ALT, AST and Alk. Phos. If more than 2 weeks have elapsed between Screen labs and Baseline (week 0), repeat Screen labs work prior to or at Baseline Visit (with the exception of bone mineral density)

** If CDI, MASC and CBCL have been completed at Screen Visit and it is < 2 weeks, these measures may not be repeated at week zero.

### Assessment Process

Screening of participants for inclusion criteria and exclusion criteria will take place at the time of initial assessment. The assessment consists of a full day, during which patients complete psychological measures (see Table 1) and undergo psychiatric, nutritional, and medical assessments. After completing a variety of psychological measures, the patient will be interviewed by members of the treating team. A psychiatrist or a psychologist along with a medical doctor will conduct the initial interview. Following the interview, the patient then completes a nutritional assessment with a registered dietician. During this time, the patient's parents are also interviewed. The medical assessment consists of a physical examination, along with documentation of vital signs (temperature plus lying and standing heart rate and blood pressure), and laboratory tests (see Table 1). After the formalized assessment is complete, determination of the patient's eligibility for the outlined study is determined.

### Inclusion Criteria

To be eligible to participate in this trial, candidates must meet the following inclusion criteria at screening:

1. Patient must be female

2. Patient and one parent must give written informed Consent or Assent.

3. Must be between ages of 12 and 17 (younger than 18) at beginning of Trial.

4. Based on the Diagnostic and Statistical Manual of Mental Disorders IV [1] must have fulfilled the criteria for diagnosis of Anorexia Nervosa (of which there are two types: restricting type or binge-eating/purging type) or Eating Disorder Not Otherwise Specified, with a Body Mass Index of less than or equal to 17.5.

### Exclusion Criteria

Candidates will be excluded from trial entry if any of the following exclusion criteria exist at screening:

1. Known sensitivity to any of the products to be administered.

2. Currently receiving treatment with any other anti-psychotic medication, mood stabilizer, or stimulant.

3. Treatment with medication known to interact with olanzapine, (eg. Fluvoxamine, Ciprofloxacin).

4. Known diagnosis of: diabetes, impaired glucose tolerance, hyperlipidemia, hepatic dysfunction, substance abuse, narrow angle glaucoma, paralytic ileus, or pancreatitis, or any other medical illness that would be considered to significantly impact treatment or recovery from AN

5. Inability to comply with trial requirements including lack of comprehension of English.

6. Pregnant or breast-feeding.

7. Other unspecified reasons that, in the opinion of the Investigator, make subject unsuitable for enrollment.

Laboratory exclusion criteria:

• Total white cell count < 2.5

• Neutrophil count < 1.0

• Liver function tests (AST/ALT > 2× normal)

• Positive pregnancy test

• EKG – QTc > 440 msec or arrythmia other than sinus bradycardia; conduction

abnormalities prolonged QTc or other

### Consent and Enrollment

When the adolescent meets inclusion and not exclusion criteria, the physician or psychiatrist will provide the patient and her family with a brief description of the study, the extent and implications of adolescent and parental involvement, and the overall purpose of the research study. The adolescent and family will be asked to consider involvement in the study and permission will be requested for a follow-up phone call from the project coordinator within the next 72 hours. As well, a meeting with the study psychiatrist will be offered during which the nature of the study will be described in greater detail and questions will be answered. The family will be reassured that they will continue to receive standard care regardless of their involvement in the study.

The project coordinator will read through the assent and consent forms with the patient and her parent(s), have all members sign, and will provide them with their first scheduled visit (see Table 1 for Schedule of Events). It is expected that most patients who consent will be enrolled into the study within two weeks of their initial assessment (screening visit). If, however, more than two weeks have elapsed since the screening visit, their baseline blood work, urinalysis, weight, height, vital signs and EKG will be repeated (but not bone mineral density).

The consent of only one parent will be required. Adolescents whose parent(s) consent to the study, but who themselves do not assent or consent, will not be included in the study and in no way will this compromise their care.

### Randomization Procedures

Participants, having provided the appropriate consent, are to be enrolled at Visit 1, after all baseline evaluations have been completed. Participants will be randomized (1:1 ratio) to olanzapine or placebo using a computer generated block-size randomization schedule. To help ensure adequate allocation concealment, the computer-generated sequence will be kept centrally at the Chalmers Research Group (CRG) on computer and can only be activated when there is an eligible participant to randomize. The research coordinator will complete an eligibility criteria checklist using a 24/7-telephone randomization system. Upon receipt of an affirmative answer of the participant eligibility, the system will release a randomization number that will correspond to a bottle of medication stored in pharmacy.

### Blinding Procedure

All personnel at the investigational site will be blinded to the treatment except for the Pharmacist (or designee). The participant and her parents will also be blinded in a similar fashion. The look-a-like placebo will be dispensed in identical bottles to the experimental medication. The research pharmacist will be responsible for labelling the medication with the participant's randomization number and placing the required dose for the 14-week treatment period. Only the pharmacist will be aware of the randomization sequence and that sequence will not be broken until analysis of the results is completed. The sequence may only otherwise be broken if requested by the independent Data Safety and Monitoring Committee (DSMC). The statistician will also be blinded to the groups when managing data sets.

### Dose Rationale

The recommended dose of olanzapine for treating psychosis in adult patients with schizophrenia is 5–20 mg/day, with doses of 30–40 mg/day not uncommon in clinical practice [27]. A study currently underway at the Ottawa Hospital (General Campus) to evaluate the efficacy and safety of olanzapine for the treatment of adults with AN used 5 mg of olanzapine as a starting dose, and increased to 10 mg/day (Dr. H. Bissada, personal communication, September, 2004).

The dosing regimen to be used for the outlined study has been derived from both current clinical practice parameters and available literature. Low doses of olanzapine (2.5–5.0 mg daily) are typically prescribed for the treatment of children and adolescents with severe eating disorders. In older or more agitated patients the dose may be increased to 7.5 mg or even 10 mg per day. These doses are typically well tolerated, with sedation as the main noted side effect. In anticipation of this trial, members of the American Academy for Eating Disorders were polled through the online email service ("AED listserv") regarding their dosing of olanzapine in clinical practice. The results of this poll revealed a wide range in the dosing of olanzapine, from 1.25–20 mg/day.

With the above guidelines in mind, olanzapine will be initiated at the lowest dose available by tablet formulation (1.25 mg). The medication will be slowly increased over a two-week period assuming patient tolerance to a maximum dose of 7.5 mg/day (1.25 mg/day for three days, followed by 2.5 mg/day for four days, 5 mg/day for one week, and then the target dose of 7.5 mg/day for 10 weeks). After a period of 10 weeks at 7.5 mg, the medication will be tapered and discontinued over a further two weeks. Table 1 outlines the exact dose schedule as part of the trial schedule.

### Psychological Measures

Patients' psychological well-being will be measured utilizing the following six scales:

Eating disorder symptoms will be measured using the **Eating Attitudes Test **(EAT-26) [28], the EAT-26 is a 26-item self-report questionnaire with a 6-point scale that measures 3 domains of disordered eating: dieting, bulimia and food preoccupation, and oral control. The EAT-26 is a widely used, well-validated instrument in this clinical population. In this study, the total EAT-26 score will be used.

Psychiatric Symptoms will be measured using the **Children's Acuity of Psychiatric Illness Scale-Child and Adolescent **(CAPI) [29], The CAPI is an outcome measure for children and adolescents that is designed to monitor change deriving from contact with mental health services. The CAPI yields a total raw score and four subscale scores, including Risk Factors, Symptoms, Functioning, and Systems support. The scale has good inter-rater reliability (.78 to .85) and good internal consistency (.87) across a variety of settings [29].

Depressive symptoms will be measured using the **Children's Depression Inventory **(CDI) [30]. The CDI is a 27-item questionnaire, which asks respondents to endorse statements about themselves reflecting cognitive, behavioural and somatic symptoms of depression. Items are rated on a 3-point scale indicating symptom severity. Total scores on the CDI can range from 0 to 52, with higher scores indicating higher levels of depressive symptomatology. The measure has been shown to have acceptable internal consistency (alphas = .71 to .89) as well as good discriminant validity when classifying children with no significant psychopathology versus depressed children [30]. The total score of the CDI will be used in this study.

Anxiety symptoms will be measured using the **Multidimensional Anxiety Scale for Children **(MASC) [31]. The MASC is a 39-item 4-point Likert-style self-report which demonstrated a satisfactory to excellent range for the intraclass correlation coefficient for the three subscales: physical symptoms, harm avoidance, and social anxiety in a sample of youth (ICC = 0.79 to 0.92) [32]. The total score on the MASC will be used in this study.

Clinicians will be asked to complete the **Eating Disorder Symptom Severity Scale **(EDS3) [33] which will examine their observations of the youth's eating disorder symptoms such as: dieting behaviours, body image preoccupations, agitation related to eating/body image, fear of fat, food anxiety, as well as overall motivation for treatment. Clinicians involved in the study have attended a workshop meant to improve inter-rater reliability for this scale (r = .75 to r = .80 was deemed acceptable)

Parents will be asked to complete the **Child Behavior Checklist **[34] in order to evaluate their perceptions of their child's mood and anxiety. The Child Behaviour Checklist (CBCL) has been demonstrated to be a reliable measure when completed by parents, with a mean alpha 0.80 being reported [34]. The CBCL is a widely used, well-validated instrument in pediatric populations.

The psychological measures will be completed at baseline, weeks 6, 12, 15 and 40 (see Table 1). The patient will complete the EAT, CDI and the MASC; the parent will complete the CBCL; and the treating physician and therapist will complete the CAPI and EDS3.

### Medical Monitoring (see Table 1 for Schedule of Events)

The baseline medical assessment will include a physical examination, monitoring of side effects/symptoms (utilizing the side-effect checklist), completion of the Acquired Involuntary Movement Scale (AIMS) to monitor extrapyramidal symptoms, taking of vital signs (temperature plus lying and standing heart rate and blood pressure), an EKG, bone mineral density, and comprehensive lab work (see Table 1). An EKG will be repeated at Weeks 2, 4, and 15. At each clinic visit, a urine sample (urinalysis and pregnancy test) will be taken, vital signs completed, and weight recorded.

The AIMS and an adverse event checklist are also completed at each clinic visit.

#### Primary Outcome Measures

The primary outcome measure will be the change from baseline on the total score of the EAT-26 score measured at week 12 prior to topping off the medication, and BMI change over the first 12 weeks of treatment.

#### Secondary Outcome Measures

Change from baseline in the EAT-26 score measured at week 6, 15 (i.e. off medication) and at the end of the maintenance period (week 40), as well as BMI change measured at the same time points will be analyzed as for the primary outcomes. Change from baseline in the CAPI, the CDI, the MASC, the EDS3 and the CBCLwill be calculated for weeks 6, 12, 15 and 40.

## Discussion

### Safety Issues

At each visit, the patient will be asked about any harm they may have experienced and clinical assessments will be performed to determine the safety of olanzapine. All adverse events will be recorded on the Case Report Form (CRF) and entered into the database. Harms include any untoward medical occurrence that a study participant experiences, whether or not that event is considered to be in relation to the study drug. Serious adverse events (SAE's) are those events that result in death or are life threatening, result in hospitalization or prolongation of hospitalization, result in persistent or significant disability, or result in a congenital birth defect. All SAE's will be reported to Health Canada as per the required reporting timelines. As well, all SAE's will be reported to an independent Data Safety and Monitoring Committee (DSMC).

If a patient endorses Item "9" "I want to kill myself" on the Child Depression Inventory (CDI), the project coordinator will immediately contact the Research Investigator and establish a plan to assess risk for suicide. The project coordinator will survey the item immediately after the adolescent completes the questionnaires.

### Sample Size

Given the relative novelty of this application of olanzapine, no formal evaluation of the drug has yet been conducted on youth, making it difficult to accurately estimate sample size requirements. However, after consultation within our clinical team we believe that a decrease of 5 points on the EAT-26 scale would be clinically meaningful. The following assumptions were therefore used for sample size calculations: i) a minimal clinical important difference of 5 on the EAT-26 score; ii) an estimated standard deviation of 7.07; iii) two-sided test; iv) a power of 80% and a type I error of 5%; v) a provision for one interim analysis according to the O'Brien and Fleming criteria [35]. With these assumptions, the total sample size necessary is 64 participants. Anticipating a drop-out rate of at most 2.5%, the formula of Donner [36] may be used to adjust the sample size assuming an intention to treat analysis. The resulting total sample size is thus 67 participants (33 and 34 participants per treatment group). We anticipate that this study will take two years to complete enrollment.

### Statistical Analysis

Descriptive summaries of demographic and treatment balance will be generated with respect to all participant characteristics at baseline. Dichotomous variables will be summarized using percentages, normally distributed continuous variables will be summarized using means together with standard deviations, and continuous variables that are not normally distributed will be summarized using medians together with range.

Statistical analysis of the data will follow the intention-to-treat principle for the main analysis of treatment outcomes. For participants who develop clinical events that will lead to withdrawal from the study, or that are lost to follow-up prior to the 12-week primary endpoint, we will use the BMI plus last value captured for the EAT-26 test as a method of imputation of missing data.

A student's t-test (assuming a normal distribution of the measures; otherwise, Wilcoxon Mann-Whitney test will be used) will be used in order to compute the change between the EAT-26 and the difference in BMI from baseline to week 12. If necessary, a linear regression model will be fit to assess treatment effect adjusting for variables thought to influence outcome that could result in imbalance between treatment groups at baseline. Treatment effect and its 95% confidence interval will be generated for each primary outcome.

To examine secondary outcomes, the differences between study groups will be assessed using Student's t-tests (as described above for primary outcome). In order to avoid multiple testing issues, results will be compared with an alpha value adjusted for the number of tests performed using the Bonferonni criterion.

Although the study is not powered to detect differences in safety, we will nevertheless compare the frequency of adverse events between the two study groups using chi-square or Fisher's exact test.

### Data Safety and Monitoring Committee

This study will be monitored by an independent DSMC formed by the steering committee. The present DSMC consists of three individuals who are not involved in this research trial: a psychologist specializing in Mental Health Research, an adolescent psychiatrist specializing in psychopharmacology, and a pediatrician specializing in drug trials (RCTs of chemotherapy in cancer patients). A statistician who is blind to the treatment allocation will conduct a tabulation of safety data, and participants' baseline characteristics after one year of recruitment. The committee will be made aware by the study coordinator of any concerning side effects, and all data will be reviewed after the enrollment of the tenth participant. Members of the DSMC will also be responsible for developing terms of reference including clinical stopping rules and examples of data presentation to be used for the tabulation of the data for the safety analysis. The steering committee will be notified by the DSMC only if the latter considers continuance of the study to be unacceptable.

### Interim analysis

One interim analysis will be run after the enrolment of the 33^rd ^patient, which will be representative of half of the sample. This analysis will focus on the primary outcome and safety issues. Using the O'Brien-Fleming criteria for two-sided test, the alpha-level of this interim analysis will be 0.005. The final analysis will be conducted using an alpha-level of 0.048.

### Reporting

The Consolidated Standards of Reporting Trials (CONSORT) statement [37,38] will be used as a guideline for the present clinical trial in order to assure high quality reporting.

## Competing interests

Eli Lilly and Company provided the olanzapine (Zyprexa) and placebo for this study. Dr. Moher is funded, in part, by a University of Ottawa Research Chair. All other authors declare that they have no competing interests.

## Authors' contributions

WS formulated the research question, contributed to the conception of the study, and helped write the study protocol. AB contributed to the conception of the study and design, and helped write the study protocol. KH, SF, KK, MN and DM contributed to the study and protocol design. DM provided expertise in the area of clinical trial methodology. IG guided the statistical methodology. SL contributed to the design and will coordinate the project. All authors have read and approved the final manuscript.

## Pre-publication history

The pre-publication history for this paper can be accessed here:


